# Improved utilization of soybean meal through fermentation with commensal *Shewanella* sp. MR-7 in turbot (*Scophthalmus maximus* L.)

**DOI:** 10.1186/s12934-019-1265-z

**Published:** 2019-12-16

**Authors:** Chaoqun Li, Beili Zhang, Xin Wang, Xionge Pi, Xuan Wang, Huihui Zhou, Kangsen Mai, Gen He

**Affiliations:** 1 Key Laboratory of Aquaculture Nutrition and Feed, Ministry of Agriculture, Ocean University of China, 5 Yushan Road, Qingdao, 266003 People’s Republic of China; 2 Key Laboratory of Mariculture (Ministry of Education), Ocean University of China, Qingdao, 266003 China; 3 Laboratory for Marine Fisheries Science and Food Production Processes, Qingdao National Laboratory for Marine Science and Technology, Qingdao, 266237 China; 4 Institute of Plant Protection and Microbiology, Zhejiang Academy of Agricultural Sciences, Hangzhou, 310021 People’s Republic of China

**Keywords:** Turbot, *Shewanella* sp. MR-7, Soybean meal, Fermentation, Intestinal health, Intestinal microbiota

## Abstract

**Background:**

Increased inclusion of plant proteins in aquafeeds has become a common practice due to the high cost and limited supply of fish meal but generally leads to inferior growth performance and health problems of fish. Effective method is needed to improve the plant proteins utilization and eliminate their negative effects on fish. This study took a unique approach to improve the utilization of soybean meal (SBM) by fish through autochthonous plant-degrading microbe isolation and subsequent fermentation.

**Results:**

A strain of *Shewanella* sp. MR-7 was isolated and identified as the leading microbe that could utilize SBM in the intestine of turbot. It was further optimized for SBM fermentation and able to improve the protein availability and degrade multiple anti-nutritional factors of SBM. The fishmeal was able to be replaced up to 45% by *Shewanella* sp. MR-7 fermented SBM compared to only up to 30% by SBM in experimental diets without adverse effects on growth and feed utilization of turbot after feeding trials. Further analyses showed that *Shewanella* sp. MR-7 fermentation significantly counteracted the SBM-induced adverse effects by increasing digestive enzymes activities, suppressing inflammatory responses, and alleviating microbiota dysbiosis in the intestine of turbot.

**Conclusions:**

This study demonstrated that plant protein utilization by fish could be significantly improved through pre-digestion with isolated plant-degrading host microbes. Further exploitation of autochthonous bacterial activities should be valuable for better performances of plant-based diets in aquaculture.

## Background

Mounting evidences during recent years have demonstrated the important roles of gut microbiota in host nutrient digestion, absorption, endocrine, metabolism and immune functions [1–3]. In particular, the genome of the gut microbiota (microbiome) provides additional metabolic capacities by contributing enzymes that are not encoded by the host genome and boost the host’s ability of dietary utilization [4]. The gut microbiota also regulates diverse aspects of cellular differentiation and metabolic processes [5–7]. These microbiota-mediated functions are highly dependent on diet and their interplays [8], while the underlying mechanisms remain elusive. The critical roles that gut microbiota appear to play have spurred research to identify functional microorganisms and their associated metabolism of dietary components.

Compared to those of terrestrial animals, the gut microbiota of fish and its functional significance are far less understood. It is known that the gut microbiota of aquatic animals is unique in many aspects. In contrast to that of human and terrestrial animals dominated by Gram-positive anaerobes, the microbial composition in the digestive tract of fish and shellfish is prevailed by Gram-negative facultative anaerobes [9]. Furthermore, the activities of microbiota in aquatic animals are greatly influenced by environmental factors such as temperature, salinity, water quality, etc. [10–12]. It is established that gut microbiota plays a key role in digestion and assimilation of terrestrial animals [13]. Similarly, the microorganisms harbored in aquatic animals may also make significant contributions to host digestion [14].

Increased inclusion of plant proteins in aquafeeds has become a common practice because of the high cost and limited supply of fishmeal [15, 16]. However, over-substitution of fishmeal with plant proteins generally leads to reduced digestion, enteritis, and inferior growth performance of fish especially carnivorous fish [17–19]. It is known that fish harbor a variety of proteolytic, amylolytic and cellulolytic bacteria [20]. However, the correlations between the host ability of plant protein utilization and the autochthonous microbes have never been well established. Better understanding the specific effects of particular autochthonous microbes and their contributions to the utilization of alternative protein sources will improve our ability to manipulate and fortify fish gut microbial communities to enhance the aquaculture productivity.

In the present study, turbot (*Scophthalmus maximus* L.), an economically valuable marine carnivorous fish with high protein requirement and sensitivity to protein sources [21], was chosen as the model species. We isolated microbes from the intestinal mucosa of the turbot (*Scophthalmus maximus* L.) through directional enrichment using soybean meal based medium. A strain of *Shewanella* sp. MR-7 was identified and its effects on soybean meal fermentation and subsequent dietary utilization by turbot were evaluated. To our knowledge, this approach has been rarely conducted.

## Results

### Isolation and identification of *Shewanella* sp. MR-7 as the leading SBM-degrading bacteria in turbot intestine

The microbiota of turbot intestine was first characterized and shown in Additional file 1: Figure S1. The SBM-degrading bacteria were enriched by culturing the isolates of turbot intestinal mucosa in the SBM liquid medium. After the culture was further inoculated onto casein agar, bacterial colonies with clear zones > 4 cm were selected and purified by dilution streaking method for further identification by 16S rDNA sequencing. Among the bacteria identified, *Shewanella* sp. MR-7 showed the best SBM degradation ability.

**Fig. 1:**
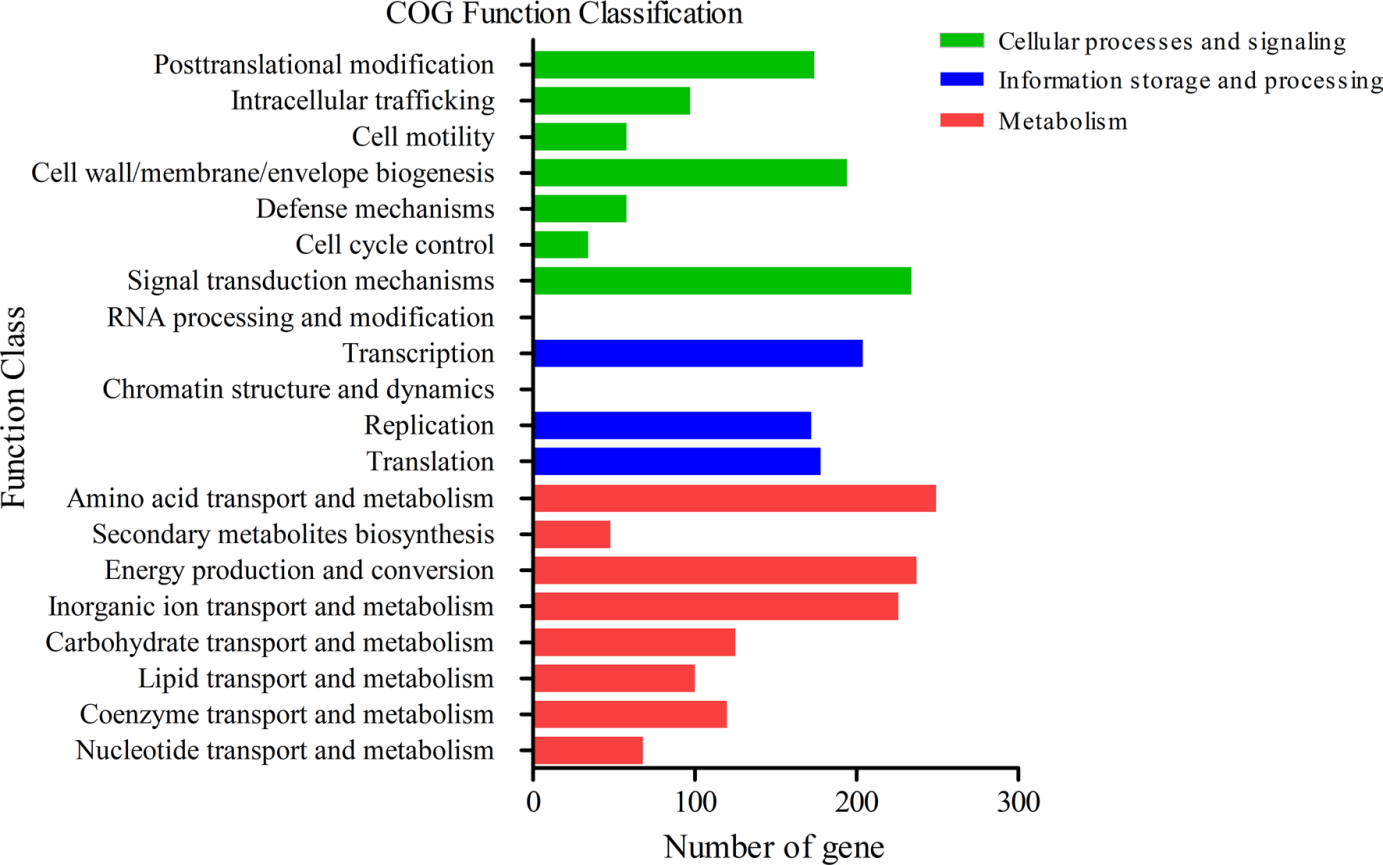
COG function classification of *Shewanella* sp. MR-7

**Fig. 2:**
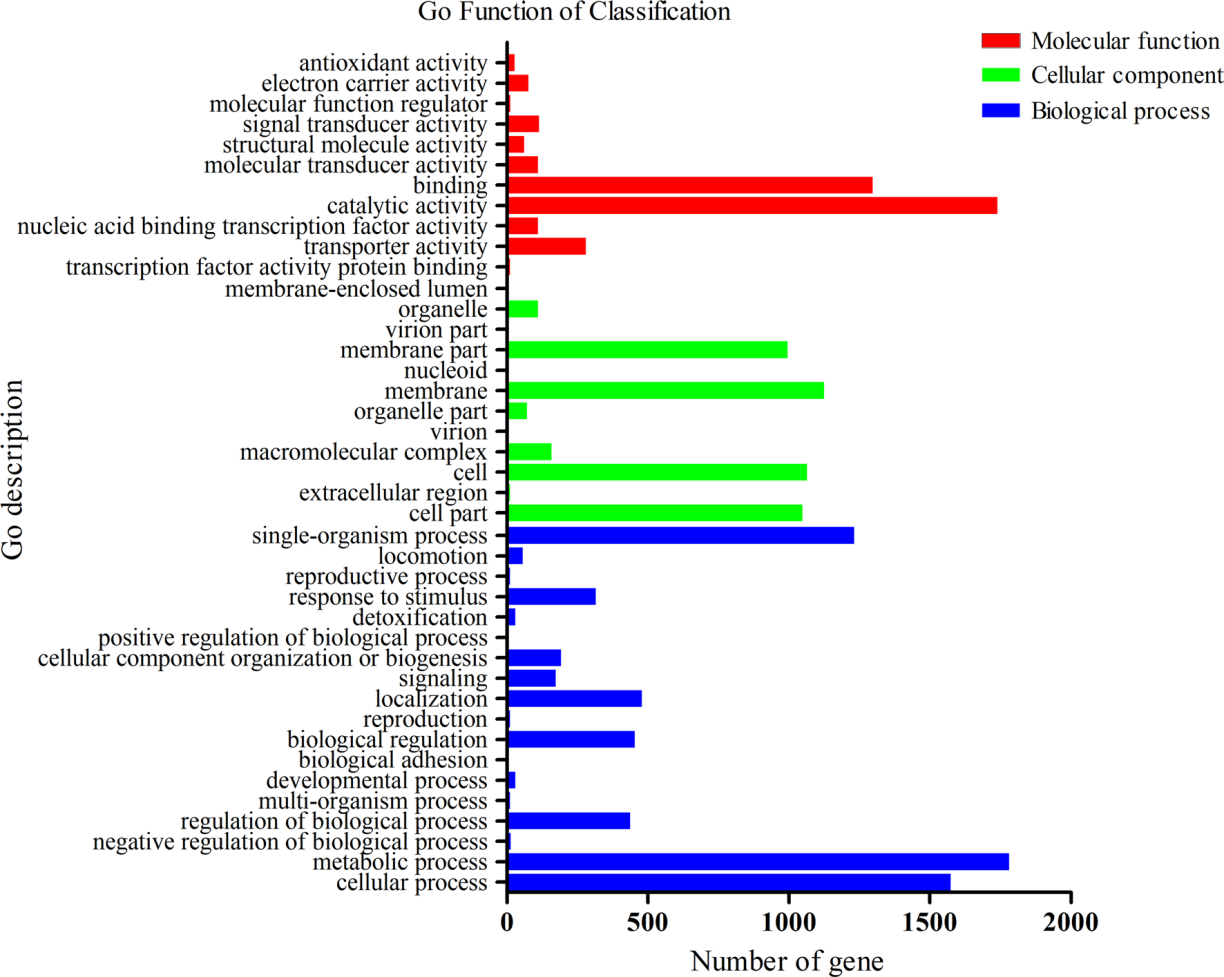
GO function annotation of *Shewanella* sp. MR-7

**Fig. 3:**
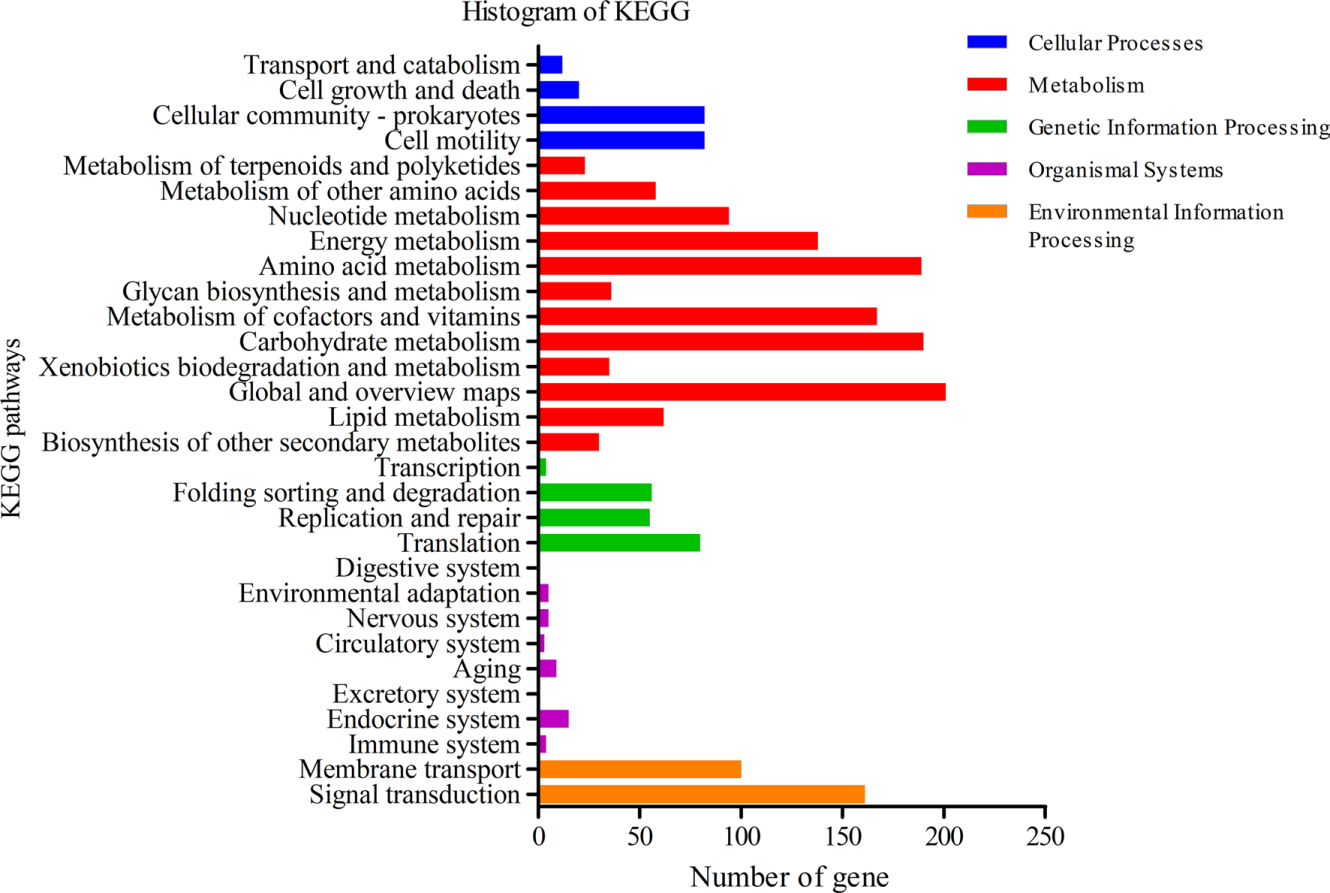
KEGG function classification of *Shewanella* sp. MR-7

The genome of *Shewanella* sp. MR-7 had a length of 4,077,231 bp and contains 4530 predicted genes with a G+C content of 48.88% (Table 1). 3709 predicted genes had COG function annotation (Fig. 1). Detailed information of the COG function annotation including genes encoding various enzymes was shown in Additional file 2: Table S2. The blast results were annotated in the GO database by blast2go and GO function annotation including cellular components, molecular functions and biological processes (Fig. 2). Moreover, 2482 predicted genes were identified to be possibly involved in 40 KEGG pathways that were classified into “metabolism”, “genetic information processing”, “cellular processes”, “environmental information processing” “organismal systems” etc. Among 1223 genes associated with “metabolism”, 190 genes were involved in carbohydrate metabolism and 189 genes were involved in amino acid metabolism (Fig. 3, Additional file 3: Table S3). Moreover, among 62 genes associated with lipid metabolism, 33 genes were involved in fatty acid (such as linoleic acid, arachidonic acid and alpha-linolenic acid) metabolism (Additional file 4: Table S4).

**Table 1:** Features of *Shewanella* sp. MR-7 genome

Attributes	Values
Gene number	4530
Gene total length	4,077,231 bp
Gene average length	900.05 bp
Gene density	0.96 genes per kb
GC content in gene region (%)	48.88
Gene/Genome (%)	85.97
Intergenetic region length	665,189 bp
GC content in intergenetic region (%)	41.75
Intergenetic length/Genome (%)	14.03

### Parameter optimization and characterization of *Shewanella* sp. MR-7

**Fig. 4:**
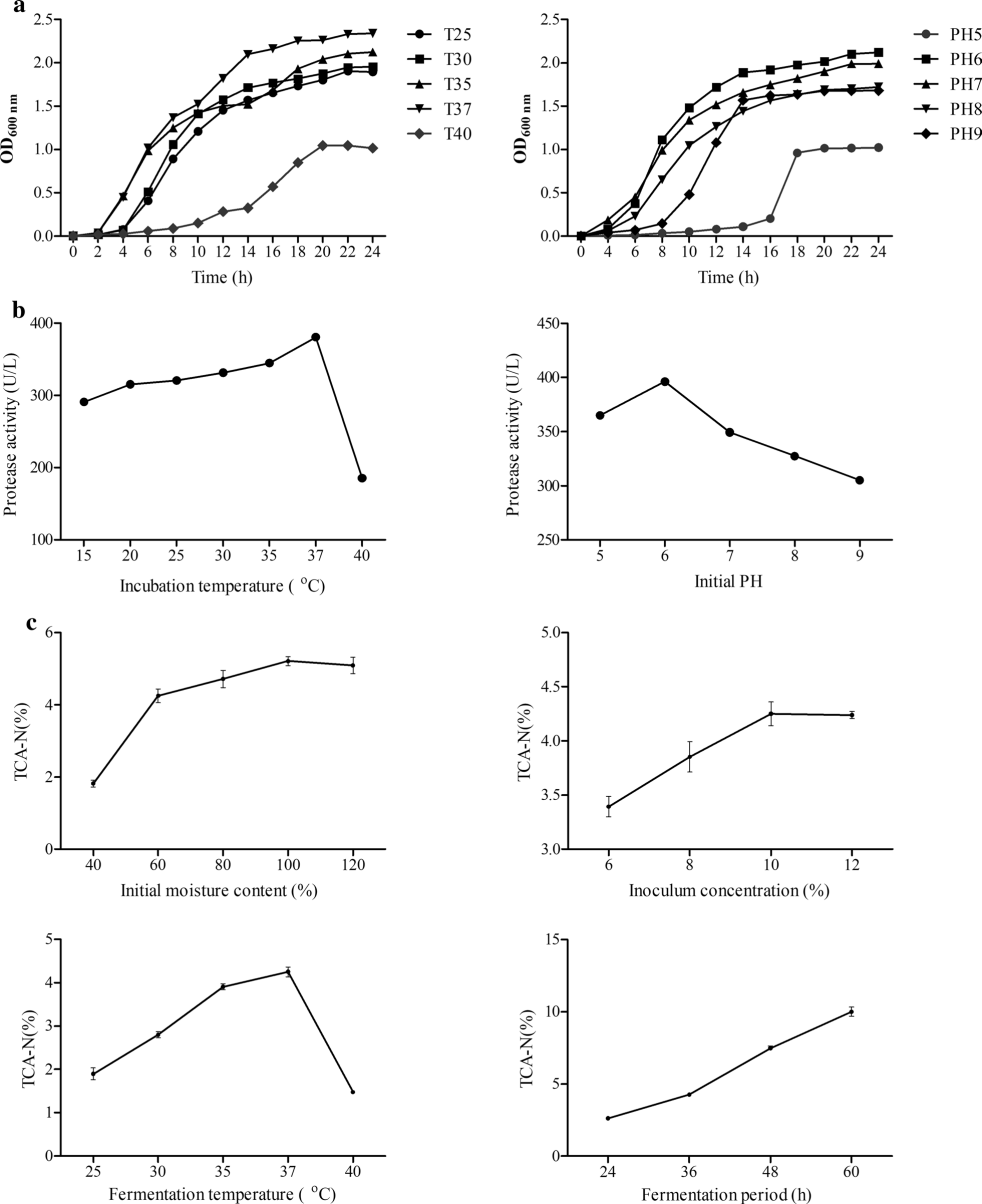
Parameter optimization and characterization of *Shewanella* sp. MR-7. **a** Growth curves of *Shewanella* sp. MR-7 under different temperatures and pH. T25, T30, T35, T37, T40: 25 °C, 30 °C, 35 °C, 37 °C, 40 °C. **b** Effects of different temperatures and pH on protease production of *Shewanella* sp. MR-7. **c** Effects of initial moisture content, inoculum concentration, fermentation temperatures and fermentation period on trichloroacetic acid soluble nitrogen (TCA-N) production under solid state fermentation

The growth of *Shewanella* sp. MR-7 under different temperature and pH was evaluated. Examined by its growth curve (Fig. 4a), the optimal growth temperature and pH for *Shewanella* sp. MR-7 was 37 °C and pH 6.0 respectively. The temperature and pH of optimal protease production by *Shewanella* sp. MR-7 were at 37 °C and pH 6.0 respectively (Fig. 4b). Under optimized condition, *Shewanella* sp. MR-7 showed secretion of several enzymes including protease (380.54 ± 0.39 U L^−1^), pectinase (18.49 ± 0.41 U L^−1^), cellulase (39.11 ± 1.23 U L^−1^), hemicellulase (93.86 ± 0.72 U L^−1^) and phytase (14.80 ± 0.40 U L^−1^).

Measured by the trichloroacetic acid soluble nitrogen (TCA-N) level (Fig. 4c), the optimal parameters of soybean meal fermentation by *Shewanella* sp. MR-7 were: initial moisture content at 100%, inoculum concentration at 10% and incubation temperature at 37 °C. Although the TCA-N production continued to increase, the possibility of contamination and unpleasant ammonia odor rose when the fermentation exceeded 60 h. Therefore, we kept the fermentation period at 48 h for further experiments. Under the optimized parameters, the *Shewanella* sp. MR-7 fermented soybean meal significantly increased the crude protein at 3.20 ± 0.42%, TCA-N at 482.79 ± 0.95% and decreased crude lipid at 21.66 ± 2.27% (Table 2). The levels of raffinose and stachyose were decreased by 29.25 ± 1.28% and 24.25 ± 1.84% respectively. The levels of anti-nutritional factors including trypsin inhibitors, glycinin and β-conglycinin were significantly decreased by 90.19 ± 0.42%, 77.36 ± 0.14% and 84.52 ± 0.18% respectively (*P* < 0.05). The digestion abilities of SBM crude protein and ANFs by *Shewanella* sp. MR-7 and other representative strains from references were compared (Additional file 5: Table S5).

**Table 2:** The nutritional profile of SBM and FSBM

Ingredients	Crude protein (%)	Crude lipid (%)	TCA-N (%)	Trypsin inhibitors (mg g^−1^)	Glycinin (mg g^−1^)	β-Conglycinin (mg g^−1^)	Raffinose (mg g^−1^)	Stachyose (mg g^−1^)
SBM	52.97 ± 0.05^a^	2.12 ± 0.02^a^	1.41 ± 0.01^a^	2.65 ± 0.05^a^	181.91 ± 0.21^a^	127.70 ± 0.46^a^	12.66 ± 0.13^a^	31.28 ± 0.25^a^
FSBM	54.70 ± 0.17^b^	1.66 ± 0.03^b^	8.24 ± 0.04^b^	0.26 ± 0.01^b^	41.19 ± 0.31^b^	19.76 ± 0.23^b^	8.96 ± 0.08^b^	23.68 ± 0.43^b^

Values show mean ± standard error, n = 3; values in the same column with different superscripted small letters mean significant difference (*P *< 0.05)

*SBM* soybean meal, *FSBM Shewanella* sp. MR-7 fermented soybean meal

### Dietary FSBM improved the growth performance and feed utilization of turbot

The performances of fermented soybean meal in the turbot diet were evaluated by a 79-day feeding trial. As shown in Table 3, no significant differences (*P* > 0.05) were found in the final body weight (FBW), specific growth rate (SGR), and weight gain rate (WGR) among groups fed with either FM diet, or diets with fishmeal levels replaced by SBM up to 30%, or by FSBM up to 45%, while all these parameters were decreased significantly in groups with higher dietary fishmeal substitution levels. The survival rate (SR) showed no significant differences among different groups (*P* > 0.05). In addition, feed intake (FI) showed no significant differences among all groups except a significant increase was found in the FSBM60 group (*P* < 0.05). The protein efficiency ratio (PER) and feed efficiency ratio (FER), as well as the apparent digestibility coefficients (ADC) of dry matter and crude protein, showed no significant reduction (*P* > 0.05) when the dietary fishmeal was replaced by SBM up to 30% and FBSM up to 45%, but reduced significantly beyond these substitution levels (*P* < 0.05).

**Table 3:** Growth parameters and feed utilization of juvenile turbot fed the experimental diets

Treatments	FBW (g)	WGR (%)	SGR (% days^−1^)	SR (%)	FER (g g^−1^)	FI (% days^−1^)	PER (g g^−1^)	ADC of dry matter (%)	ADC of crude protein (%)
FM	73.92 ± 0.90^a^	8.79 ± 0.13^a^	2.89 ± 0.01^a^	98.89 ± 1.11	1.34 ± 0.05^a^	1.55 ± 0.06^a^	2.90 ± 0.12^a^	57.41 ± 0.40^a^	93.07 ± 0.09^a^
SBM15	69.78 ± 1.64^a^	8.21 ± 0.22^a^	2.81 ± 0.03^a^	96.67 ± 0.00	1.29 ± 0.01^ab^	1.58 ± 0.01^ab^	2.63 ± 0.06^ab^	55.59 ± 0.55^a^	92.31 ± 0.40^a^
SBM30	69.38 ± 1.60^a^	8.15 ± 0.21^ab^	2.80 ± 0.03^a^	96.67 ± 1.92	1.21 ± 0.02^abc^	1.65 ± 0.01^ab^	2.60 ± 0.13^ab^	54.87 ± 0.38^a^	91.93 ± 0.11^a^
SBM45	56.96 ± 1.68^c^	6.52 ± 0.22^c^	2.55 ± 0.04^c^	96.67 ± 1.92	1.18 ± 0.01^bc^	1.63 ± 0.04^ab^	2.44 ± 0.07^b^	48.31 ± 0.67^bc^	89.13 ± 0.37^b^
SBM60	55.47 ± 1.28^c^	6.31 ± 0.17^c^	2.52 ± 0.03^c^	97.78 ± 1.11	1.23 ± 0.06^bc^	1.57 ± 0.08^ab^	2.43 ± 0.07^b^	45.84 ± 0.64^c^	86.94 ± 0.19^c^
FSBM15	70.00 ± 2.06^a^	8.24 ± 0.28^a^	2.81 ± 0.04^a^	95.56 ± 1.11	1.24 ± 0.03^abc^	1.61 ± 0.04^ab^	2.64 ± 0.03^ab^	57.10 ± 0.31^a^	93.02 ± 0.18^a^
FSBM30	73.03 ± 1.35^a^	8.65 ± 0.17^a^	2.87 ± 0.02^a^	95.56 ± 2.22	1.30 ± 0.02^ab^	1.59 ± 0.03^ab^	2.66 ± 0.04^ab^	55.48 ± 0.59^a^	92.96 ± 0.20^a^
FSBM45	68.92 ± 2.00^ab^	8.10 ± 0.26^ab^	2.79 ± 0.03^ab^	96.67 ± 1.92	1.27 ± 0.02^ab^	1.60 ± 0.02^ab^	2.62 ± 0.03^ab^	55.39 ± 0.63^a^	92.07 ± 0.11^a^
FSBM60	59.47 ± 3.84^bc^	6.86 ± 0.50^bc^	2.60 ± 0.08^bc^	96.67 ± 1.92	1.12 ± 0.03^c^	1.75 ± 0.02^b^	2.33 ± 0.06^b^	50.75 ± 0.43^b^	88.84 ± 0.57^b^

Values show mean ± standard error, n = 3; values in the same column with different superscripted small letters mean significant difference (*P *< 0.05)

*FBW* final body weight, *WGR* weight gain rate, *SGR* specific growth rate, *SR* survival rate, *FER* feed efficiency ratio, *FI* feed intake, *PER* protein efficiency ratio, *ADC* apparent digestibility coefficients

### Dietary FSBM increased the intestinal digestive enzyme activities

As shown in Table 4, compared to that of the FM group, the trypsin activity was decreased significantly when the dietary fishmeal substitution by SBM ≥ 30% or by FSBM ≥ 60% (*P* < 0.05). The lipase activity increased in FSBM15 and FSBM 30 groups or remained similar in FSBM45 and FSBM60 groups, but decreased significantly in all SBM groups, when compared to that of the FM group. The intestinal diastase activity of turbot showed no significant differences among groups except significant reductions in SBM45 and SBM60 groups.

**Table 4:** Activity of intestinal digestive enzyme of juvenile turbot fed the experimental diets

Treatments	Trypsin (10 U mg^−1^)	Diastase (U mg^−1^)	Lipase (U mg^−1^)
FM	39.67 ± 1.71^ab^	0.20 ± 0.01^a^	47.14 ± 0.98^bc^
SBM15	35.24 ± 0.65^bc^	0.21 ± 0.01^a^	39.17 ± 1.18^d^
SBM30	32.38 ± 0.59 ^cd^	0.20 ± 0.01^a^	38.45 ± 0.52^d^
SBM45	30.51 ± 0.42 ^cd^	0.15 ± 0.01^bc^	32.91 ± 1.05^d^
SBM60	26.83 ± 2.01^d^	0.13 ± 0.01^c^	23.98 ± 0.66^e^
FSBM15	39.50 ± 1.17^ab^	0.22 ± 0.01^a^	57.07 ± 1.63^a^
FSBM30	42.52 ± 0.65^a^	0.21 ± 0.01^a^	56.61 ± 1.50^a^
FSBM45	41.16 ± 1.45^a^	0.19 ± 0.01^ab^	52.28 ± 2.13^ab^
FSBM60	28.40 ± 0.93^d^	0.19 ± 0.01^ab^	40.46 ± 2.90 ^cd^

Values show mean ± standard error, n = 6; values in the same column with different superscripted small letters mean significant difference (*P *< 0.05)

### Dietary FSBM suppressed the intestinal inflammation and enhanced the intestinal integrity

**Fig. 5:**
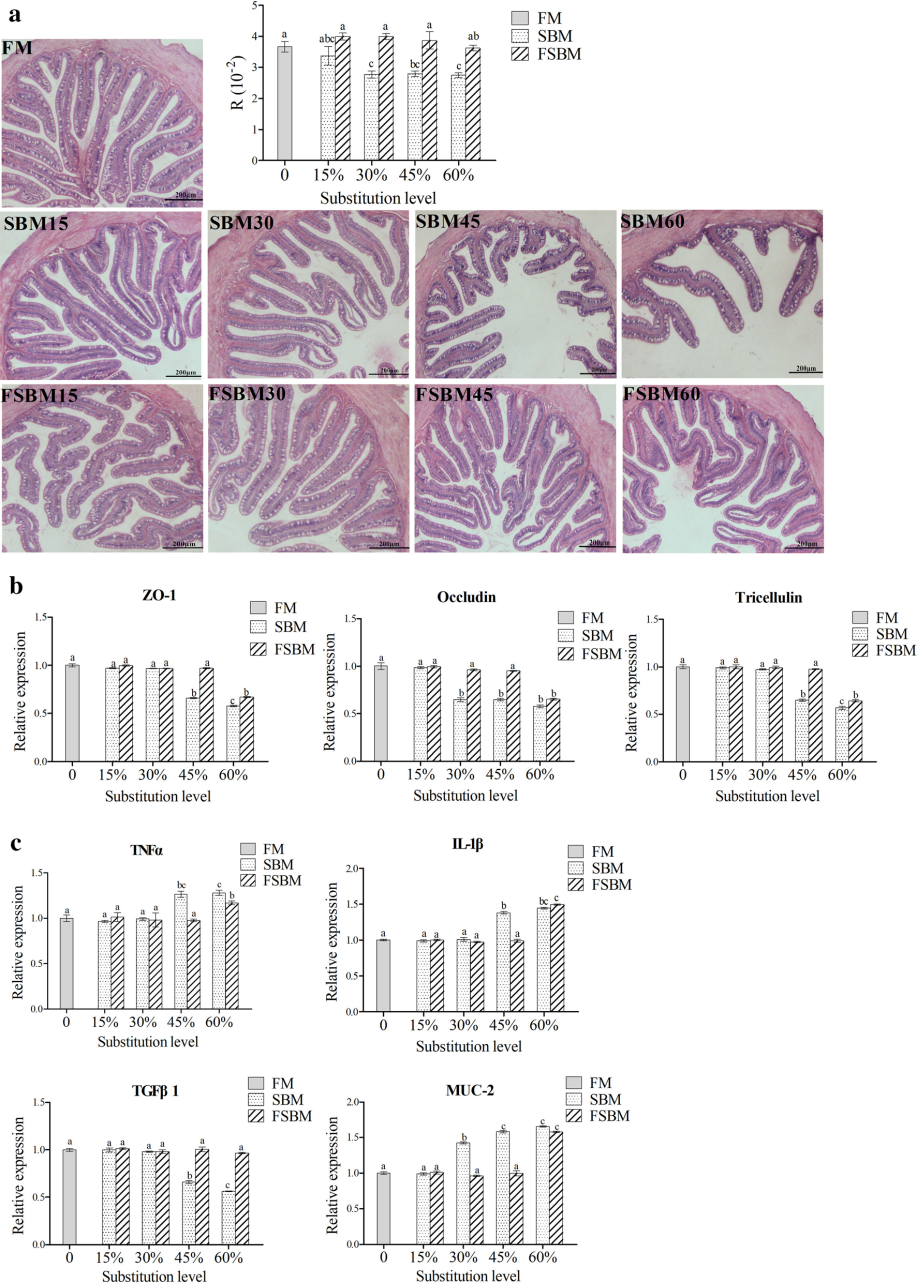
The intestinal morphology, integrity, and inflammation status of turbot fed experimental diets. **a** Representative histomorphological images from hematoxylin and eosin-stained sections and the villi height analysis of the distal intestine of turbot. **b** Effects of experimental diets on intestinal integrity marker gene expressions of turbot. **c** Effects of experimental diets on intestinal inflammation marker gene expressions of turbot. The value of FM group was normalized to 1.0 and the rest groups were expressed as relative expression levels to the FM group. Values show mean ± standard error (n = 6) and different letters (a, b, c) on top represent statistically significant results (*P* < 0.05) based on one-way analysis of variance (ANOVA) by the software SPSS 19.0. *R* villi height/lumen diameter, arbitrary units, *TNF-α* tumor necrosis factor-α, *IL-1β* interleukin-1β, *TGF-β1* transforming growth factor-β1, *MUC-2* mucin 2, *ZO-1* zonula occluden-1

As shown in Fig. 5a, with the increases of fishmeal replacement by SBM, the height and number of mucosal folds were reduced in the distal intestine. The length of microvilli was also decreased steadily with the increased inclusion of SBM levels. However, no significant morphological changes were observed in the distal intestines of FSBM groups compared with those of the FM group. Furthermore, the expression levels of tight junction genes of occludin, ZO-1 transcript variant 1 and tricellulin were significantly reduced in SBM groups with dietary fishmeal replacement ≥ 30%, ≥ 45%, ≥ 45% respectively (Fig. 5b). However, the expression levels of these genes were only reduced in FSBM groups with dietary fishmeal replacement ≥ 60% (Fig. 5b). In addition, the expression levels of pro-inflammatory (IL-1β and TNF-α) and anti-inflammatory (TGF-β1) cytokines were significantly increased or decreased respectively in SBM groups with dietary fishmeal replacement ≥ 45% (Fig. 5c). However, only the expression levels of prof-inflammatory cytokines in FSBM60 showed increases among the FSBM groups, while that of the TGF-β1 remained stable among all the FSBM groups. The expression level of MUC-2, the major secretory mucin and biomarker of intestine inflammation [22] was increased significantly in SBM groups with dietary fishmeal replacement ≥ 30%, while only that of FSBM60 showed significant increase in fermented groups (Fig. 5c).

### Fermentation alleviated SBM induced intestine microbiota dysbiosis

**Fig. 6:**
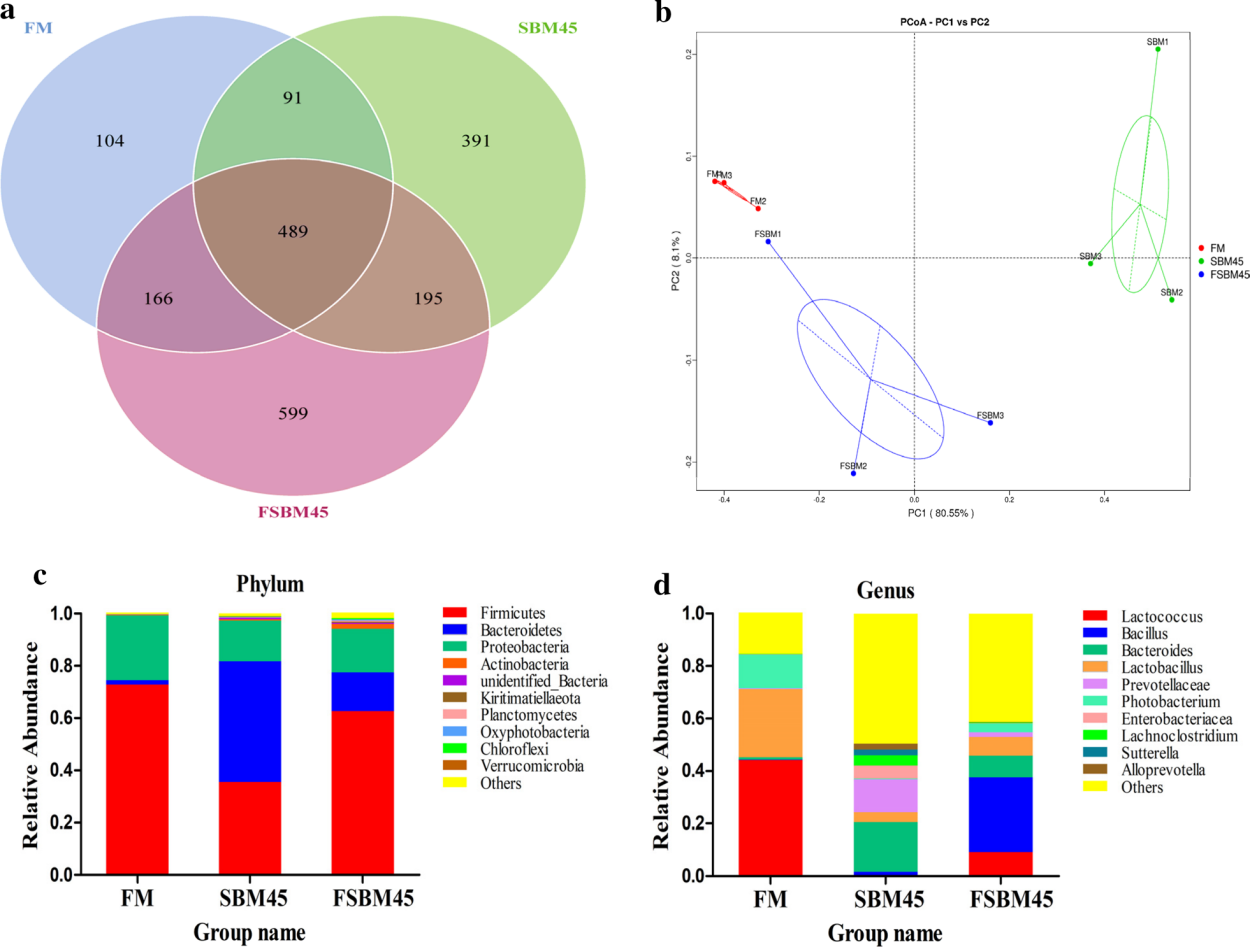
Intestinal mucosal microbiome composition of turbot fed FM, SBM45 and FSBM45 diets. **a** Venn diagram of unique and shared OTUs. **b** Principal co-ordinates analysis (PCoA) for the autochthonous bacterial communities of turbot. **c** Taxonomy classification of reads at phylum taxonomic levels. **d** Taxonomy classification of reads at genus taxonomic levels. Only top 10 most abundant (based on relative abundance) bacterial phyla or genera were shown in the figures. Other phyla or genera were all assigned as ‘Others’. FM1, FM2 and FM3 are three replicates of FM group; SBM1, SBM2 and SBM3 are three replicates of SBM45 group; FSBM1, FSBM2 and FSBM3 are three replicates of FSBM45 group

To analyze the possible intestine microbiome changes, we select the FM, SBM45 and FSM45 as the representative experimental groups for detailed examinations. After sequence quality control and feature filtering, a total number of 650,776 clean reads were generated, covering 5437 OTUs (97% similarity level) from 9 samples in FM, SBM45 and FSBM45 groups. The rarefaction curves for observed species number tended to approach the saturation plateau, indicating adequate sequencing depth for all samples (Additional file 5: Figure S2). A Venn diagram showed that 489 OTUs were shared by FM, SBM45 and FSBM45 groups, and the number of unique OTUs in group FM, SBM45 and FSBM45 was 104, 391 and 599, respectively (Fig. 6a). The changes of bacterial richness (expressed by Chao1 and ACE) and diversity (expressed by Shannon and Simpson) in response to FM, SBM45 and FSBM45 were shown in Table 5. The Chao1 index and ACE index were significantly increased in FSBM45 compared to those of FM and SBM45 groups (*P* < 0.05). The alpha-diversity indicated by Shannon index and Simpson index were significantly increased in FSBM45 and SBM45 compared to that of FM (*P* < 0.05). Moreover, the PCoA plot showed clear clusters of microbial samples from different treatments, indicating a different response model of intestinal microbiota to various diets (Fig. 6b). The FSBM clusters were found to be more coherent with FM clusters than SBM clusters (Fig. 6b).

**Table 5:** Alpha diversity index of intestinal microbiota of experimental turbot

Treatments	Richness estimates		Diversity estimates	
	Chao1	ACE	Shannon	Simpson
FM	702.38 ± 24.07^a^	744.68 ± 19.30^a^	3.39 ± 0.30^a^	0.77 ± 0.03^a^
SBM45	694.79 ± 22.30^a^	690.11 ± 19.18^a^	5.59 ± 0.34^b^	0.94 ± 0.01^b^
FSBM45	851.85 ± 28.17^b^	866.88 ± 40.26^b^	5.36 ± 0.31^b^	0.89 ± 0.02^b^

As shown in Fig. 6c and Additional file 6: Table S6, the intestine mucosal microbiota of FM and FSBM45 were dominated by Firmicutes (72.86%, 62.56%). However, the intestine mucosal microbiota of SBM45 were dominated by Bacteroidetes (46.20%), followed by Firmicutes (35.581%). Compared with FM, dietary SBM significantly reduced the relative abundances of Firmicutes by more than 2 times (*P* < 0.05) and increased the relative abundance of Bacteroidetes by more than 25 times (*P* < 0.05). However, *Shewanella* sp. MR-7 fermentation alleviated these SBM-induced changes and showed more similarity to bacterial composition of FM group. At genus level, *Lactococcus*, *Bacillus*, *Bacteroides*, *Lactobacillus*, *Prevotellaceae*, *Photobacterium*, *Enterobacteriaceae*, *Lachnoclostridium*, *Sutterella* and *Alloprevotella* formed the top ten dominant genera, and their relative abundances were significantly influenced by dietary treatment (*P* < 0.05) (Fig. 6d and Additional file 7: Table S7). *Lactococcus* was accounted for 44.19% of the total bacterial population in FM, followed by *Lactobacillus* (26.01%). *Bacillus* was accounted for 28.57% of the total bacterial population in FSBM, followed by *Lactococcus* (9.08%). *Bacteroides* was accounted for 18.88% of the total bacterial population in SBM, followed by *Prevotellaceae* (9.08 ± 0.59%). Dietary SBM significantly increased the relative abundances of *Bacteroides*, *Prevotellaceae*, *Enterobacteriaceae* and significantly reduced the relative abundances of *Lactococcus* and *Lactobacillus* compared to those of control. However, *Shewanella* sp. MR-7 fermentation alleviated these SBM-induced changes.

## Discussion

Gut microbiota play an important role in food digestion, nutrient absorption, metabolism and immune of host aquatic animals [23]. Previous studies have isolated various fish-derived probiotics such as *Weissella hellenica DS*-*12* from Japanese flounder [24], *Lactobacillus fructivorans AS17B* from sea bream [25], *Lactococcus lactis WFLU12* from olive flounder [26] and confirmed their functions in immune responses and disease control. However, few studies examined the roles of host-derived strains on digestion and nutritional physiology. In this study, a *Shewanella* sp. MR-7 strain was isolated from the intestinal mucosa of the turbot through directed enrichment using soybean meal (SBM) based medium. Its genetic, metabolic, and fermentation properties were characterized. The performances of its SBM fermented product was evaluated by growth, nutrition utilization, gut health and gut microbiome in turbot after a feeding trial.

*Shewanella*, a genus created by MacDonell and Colwell, is phylogenetically affiliated to the γ-Proteobacteria [27]. *Shewanella* genus was detected in turbot intestine in the present study (Additional file 1: Figure S1) as well as in previous studies [28, 29]. However, *Shewanella* was not among the top 10 most abundant genera (Fig. 6d and Additional file 7: Table S7), suggesting that plant protein degrading microbes such as *Shewanella* sp. MR-7 was not dominant in carnivorous fish like turbot, and could only be isolated through directed enrichment using plant protein based culture medium.

Several studies have been done to explore the growth characteristics of *Shewanella*. The optimal growth of *Shewanella marisflavi* SW-117 and *Shewanella aquimarina* SW-120 occurred at 30–37 °C, pH 7–8 [30], *Shewanella waksmanii* KMM 3823 at 20–22 °C, pH 7.5 [31], *Shewanella colwelliana* ATCC 39565 at 25 °C, pH 7.4–7.8 [32]. In this study, *Shewanella* sp. MR-7 gained the optimal growth under the cultural condition of 37 °C and pH 6.0. These results demonstrated that the growth performance varied among different species in the genus *Shewanella* due to the diversity of the species.

It has been reported that gut microbiota might have beneficial effects on the digestion of fish by contributing various enzymes [20]. Most of the enzyme-producing bacteria isolated from the fish intestine so far belong to the genus *Bacillus* [33–35]. However, the enzyme-producing capacity of *Shewanella* was rarely characterized. In the present study, the strain *Shewanella* sp. MR-7 showed substantial production of enzymes including protease, pectinase, cellulase, hemicellulase and phytase. A considerable amount of genes in the genome of *Shewanella* sp. MR-7 were coded for carbohydrate and protein metabolism, suggesting its potential ability of plant protein utilizations.

Compared to SBM, fermented SBM (FSBM) by fungal and bacterial strains (predominantly *Aspergillus oryzae* and *Lactobacillus subtilis*, respectively) showed numerous benefits including degradation of anti-nutritive compounds and improvement of nutritional value of SBM [36]. In this study, trypsin inhibitor, glycinin and β-conglycinin in SBM were decreased by 90.19%, 77.36% and 84.52% respectively after *Shewanella* sp. MR-7 fermentation. We previously showed that SBM fermentation by *Lactobacillus plantarum* P8 degraded trypsin inhibitor, glycinin and β-conglycinin at 87%, 78% and 55% respectively [37]. Another study found that 50%, 58% and 70% of TI, glycinin subunit and β-conglycinin were removed respectively after fermentation by *B. subtilis* KCCM11438P [38]. SBM contains oligosaccharides (raffinose and stachyose) that cannot be utilized by monogastric animals [39]. In this study, the raffinose and stachyose in SBM were reduced by 29.25% and 24.25% respectively after fermentation. A previous study indicated stachyose in SBM was decreased by 83.9% after fermentation with *Rhizopus oligosporus* [40]. In another study, *B. amyloliquefaciens* U304 and *S. cerevisiae* CJ1697 drastically decreased the levels of raffinose and stachyose, while *Lactobacillus* spp. showed no effects against these oligosaccharides [41]. Fermentation is also able to degrade proteins and carbohydrates to low molecular weight and water-soluble compounds, which will facilitate nutrient digestibility and bioactivity [42]. Trichloroacetic acid soluble nitrogen (TCA-N) is an important index reflecting the small peptide and free amino acid content in fermented soybean meal [40]. The TCA-N was increased by 482.79 ± 0.95% after *Shewanella* sp. MR-7 fermentation, which was superior to previous report showed that the TCA-N in SBM could be increased by 69% after *Aspergillus oryzae* fermentation [43]. Table S3 summarized the comparisons of quality parameters of FSBM by different strains. As shown above, the fermented products vary in levels of nutritional components because of the differential metabolic activities of the microbial strain used. *Shewanella* sp. MR-7 fermentation significantly improved the quality of SBM and was comparable and even superior to a lot of strains currently used for SBM fermentations.

Along with our previous studies [37], we showed that SBM could replace up to 30% of fishmeal without adverse effects in turbot diet. In the present study, FSBM by *Shewanella* sp. MR-7 improved the fishmeal replacement level to 45% without influencing the growth performance of the turbot. Suppression of feed intake (FI), poor nutrient digestibility and presence of ANFs have been considered the major limitations of plant protein usages in aquaculture [44, 45]. In the present study, dietary FSBM did not suppress the FI and enhanced the apparent digestibility coefficients (ADC), which were lower at the same inclusion levels of the SBM. As discussed above, *Shewanella* sp. MR-7 also showed superior abilities of degrading multiple ANFs in SBM. The significantly improved quality of FSBM and enhanced ADC values of FSBM diets could be partially account for the better growth performance of turbot in FSBM groups.

The activity of intestinal digestive enzymes is an important indicator reflecting the ability of feed digestion [46]. In parallel with the aforementioned findings in ADC values, dietary FSBM also remarkably ameliorated SBM-induced decrease of the intestinal digestive enzymes (trypsin, lipase and diastase) activities. It was known that ANFs in SBM could inhibit digestive enzyme activities thereby affecting the efficiency of the digestive process [47]. Previous studies also suggested that gut microbiota contributed host digestions by producing various enzymes [20]. In the present study, the better profile of digestive enzymes in FSBM groups might be due to degradation of ANFs and modulation of gut microbiota by dietary FSBM diets.

The present study, as well as previous reports, demonstrated that high inclusion of SBM caused enteritis, which was a major contributor of the inferior performance of the diets [18]. This was further confirmed by the reduced expression levels of intestinal tight junction proteins including ZO-1, tricellulin, and occludin. Similar results were observed in Atlantic salmon treated with SBM diets [48–50]. Furthermore, the expression levels of pro-inflammatory (IL-1β and TNF-α) and anti-inflammatory (TGF-β1) cytokines were significantly increased or decreased respectively in SBM groups with dietary fishmeal replacement ≥ 45%. Mechanistically, IL-1β and TNF-α could influence the intestinal barrier through reducing the levels of ZO-1 and occludin via MLCK signaling pathway [51, 52]. Mediated through NF-κB and P13K/Akt signaling pathways, these pro-inflammatory responses could also stimulate the level of MUC-2 [53–55], which was also a biomarker for intestinal inflammation [22] and up regulated in SBM groups. Actually, the antigenic components of glycinin and β-conglycinin in SBM may be primarily accounted for the gut pathological alterations and detrimental inflammatory response [56, 57]. In contrast, judged from histology, the expression of above pro-inflammatory and tight junction factors, the intestines of FSBM groups remained healthy except some inflammatory response of FSBM60. In the present study, most of the glycinin (77.36%) and β-conglycinin (84.52%) in SBM were degraded by *Shewanella* sp. MR-7 fermentation, which could be responsible for the improved gut health of turbot fed FSBM diets. In addition, recent study indicated that soya-saponins induced enteritis and compromised the intestinal barrier functions in turbot [58]. The possibly degrading soya-saponins in FSBM may partially be accounted for the improved gut health that study on the quantitative analyses of soya-saponins contents in SBM and FSBM could be performed in the future.

Numerous studies have suggested that a healthy microbiota is critical for gut function and health [59–61]. Microbial dysbiosis is involved in various diseases including chronic enteritis in humans and mammals [62, 63]. The intestine mucosal microbiota of turbot was dominated by Firmicutes and Proteobacteria, which was in line with previous study [64]. Dietary inclusion of SBM increased the bacteria diversity, changed the relative abundance of the dominant taxon and genus, and resulted in a significantly different microbial community from that of control group, which was FM fed. Similar results were obtained in previous studies on turbot [65] and other carnivorous fish [66, 67], suggesting that dietary plant protein source may increase diversity and alter the structure of the intestinal autochthonous bacteria communities. Specifically, the microbiota of SBM45 group showed significantly increased abundances of *Prevotellaceae* and *Enterobacteriaceae*, some of which were considered as potentially colitogenic bacteria in humans and mammals [68, 69]. However, the relative abundances of the recognized probiotics, *Lactococcus* and *Lactobacillus* [70], were significantly decreased in SBM45 group compared to control. Few studies have been done to explore the effects of dietary fermented soybean meal on gut microbiome of carnivorous fish. Our data demonstrated that *Shewanella* sp. MR-7 fermentation could significantly alleviate the SBM-induced gut microbiota dysbiosis by enhancing potentially probiotic and inhibiting pathogenic bacteria.

## Conclusions

In the present study, the isolated intestinal autochthonous microbe, *Shewanella* sp. MR-7, could greatly improve the protein availability and degrade multiple anti-nutritional factors of SBM by fermentation. *Shewanella* sp. MR-7 fermentation significantly counteracted the SBM-induced adverse effects by increasing digestive enzymes activities, suppressing inflammatory responses, and alleviating microbiota dysbiosis in the intestine of turbot. This study confirmed the role of specific intestinal autochthonous microbe in the expansion of host metabolic capacity and provide clues for discovery and development of new fermentation strains.

## Methods

### Isolation and identification of proteolytic bacteria from turbot

Healthy turbots (~ 500 g in weight) were obtained from Yihaifeng fish farm Inc. Intestinal mucosa samples were collected and handled as previously described with modifications [66, 71]. Specifically, intestines were aseptically dissected and intestinal mucosa were scraped and collected in sterile tubes. All samples were serially diluted with sterile artificial seawater and inoculated into skimmed milk powder medium at 22 °C to reach the OD value > 0.5, thus enriching the proteolytic bacteria [72]. Subsequently, 100 μL of the cultured solution was inoculated into soybean meal (SBM) liquid medium (SBM, 5.0 g L^−1^; sea salt, 36.0 g L^−1^) at 22 °C to reach OD value > 0.5. The cultured solutions were serially diluted every tenfold and inoculated onto casein agar (1% casein in 5 M NaOH and sea salt medium at 1:1 with 1.5% agar) to screen out the strains with superior protease-producing ability [73]. The plates were incubated at 22 °C and detected if clear zone would be produced. Colonies producing a halo with diameter exceeding 4 cm were selected and purified by dilution streaking method. All the purified colonies were further identified based on biochemical tests and 16S rRNA gene sequencing. Moreover, the selected clones were inoculated into the SBM liquid medium for 48 h at 22 °C. The supernatant of un-inoculated and inoculated SBM liquid medium were collected by centrifuging at 10,000 rpm for 1 min. The soluble protein content in supernatant was measured using Folin-Phenol method [74].

### Genomic sequencing and bioinformatic analyses

For genomic sequencing and bioinformatic analyses, the bacterial DNA was extracted and purified using the Wizard^®^ Genomic DNA Purification Kit (Promega). The quantity of the DNA was measured using a TBS-380 fluorometer (Turner BioSystems Inc., Sunnyvale, CA). High quality DNA (OD260/280 = 1.8–2.0, > 1 μg) was used in sequencing library construction using the NEXTflex™ Rapid DNA-Seq Kit. The library was PCR amplified and used for paired-end Illumina sequencing (2 × 150 bp) on an Illumina HiSeq X Ten machine. Low quality data in the raw reads was removed to form clean data by a statistic of quality information for quality trimming, and the clean reads were assembled by SOAPdenovo v2.04. The inner gaps that emerged in the scaffold were filled using the Gap Closer version 1.12. The scaffolds were then uploaded to the CGView Server to plot the graphical circular genome map [75]. The coding sequences (CDS) were predicted using Glimmer version 3.02 and annotated from the Clusters of Orthologus Groups (COG), Kyoto Encyclopedia of Genes and Genomes (KEGG), and Gene Ontology (GO) databases [76] using sequence alignment tools such as BLAST, Diamond and HMMER. tRNA and rRNA sequences were predicted by tRNA-scan-SE (v1.2.1) [77] and RNAmmer (v1.2) [78], respectively.

### Parameter optimization of *Shewanella* sp. MR-7

The isolated strain of *Shewanella* sp. MR-7 was cultured in 2216E medium (Qingdao Haibo) and shaken at 220 rpm at 25, 30, 35, 37 and 40 °C to determine the effects of temperature on the growth of *Shewanella* sp. MR-7. The *Shewanella* sp. MR-7 was also cultured in 2216E medium at pH 5.0, 6.0, 7.0, 8.0, 9.0 to examine the effect of pH on its growth. The OD_600_ of the culture was taken periodically. The influences of temperature and pH on the production of protease by *Shewanella* sp. MR-7 were determined by enzyme-linked immunosorbent assay (ELISA) [79] using protease ELISA kits (HengYuan Biological Technology Co., Ltd, Shanghai, China).

To optimize the solid state fermentation parameters of *Shewanella* sp. MR-7 for soybean meal, *Shewanella* sp. MR-7 grown in 2216E liquid medium was inoculated with soybean meal with series levels of initial inoculum (6%, 8%, 10%, 12%), moisture (40, 60, 80, 100 and 120%), temperature (25, 30, 35, 37 and 40 °C), and fermentation period (24 h, 36 h, 48 h and 60 h). The corresponding optimal parameters were determined respectively by quantification of trichloroacetic acid soluble nitrogen (TCA-N) production under solid state fermentation. All experiments were carried out in triplicates. The TCA-N production was determined by a modified method according to the previous study [43]. Briefly, approximately 3.0000*g* of sample was placed in a 250 mL conical flask, 100 mL of 15% trichloroacetic acid solution was added and the mixture was shaken for 30 min at room temperature. After standing for 5 min, the supernatant was collected and centrifuged at 4800*g* for 10 min. The nitrogen content in final supernatant was determined by Kjeldahl method [80].

### Soybean meal fermentation and experimental diet preparation

The soybean meal was fermented using parameters optimized above with minor modifications. Briefly, SBM soaked with 100% distilled water (contained 2.6‰ sea salt, 3.3‰ (NH_4_)_2_SO_4_, 1.3‰ glucose) was autoclaved at 105 °C for 20 min in a steam tank (model HX14G-1, Shanghai, China) and cooled to room temperature. Thereafter, the SBM were inoculated with 10% of *Shewanella* sp. MR-7 (~ 10^8^ colony forming units (cfu) mL^−1^) and fermented in an incubator at 37 °C for 48 h. The resulting FSBM was dried in an oven at 45 °C until its moisture content was below 10%. The FSBM samples were collected for nutritional profiling.

The composition of experimental diets was shown in Table 6. Nine isonitrogenous (approximately 50% crude protein) and isoenergetic (approximately 20.0 kJ g^−1^ diet of gross energy) diets were formulated with 0% (FM), 15% (SBM15/FSBM15), 30% (SBM30/FSBM30), 45% (SBM45/FSBM45) and 60% (SBM60/FSBM60) of protein from FM replaced by SBM or FSBM respectively. Lysine and methionine were supplemented in all diets to the level of FM diet to meet the essential amino acid requirements of juvenile turbot. Y_2_O_3_ (0.1%) was supplemented as the indicator for the apparent digestibility determination. Experimental diets were made following the previous procedure [21] using a pellet-making machine (F-26 (II), South China University of Technology).

**Table 6:** Formulas and proximate composition of the experimental diets (% dry matter)

Ingredients	Treatments								
	FM	SBM15	SBM30	SBM45	SBM60	FSBM15	FSBM30	FSBM45	FSBM60
Fish meal^a^	60.00	51.00	42.00	33.00	24.00	51.00	42.00	33.00	24.00
Soybean meal^a^	–	12.34	24.69	37.03	49.37	–	–	–	–
Fermented soybean meal^b^	–	–	–	–	–	11.95	23.90	35.86	47.81
Wheat meal^a^	24.94	18.00	13.46	8.30	3.48	18.49	14.06	9.77	4.81
Wheat gluten meal^a^	2.46	3.73	4.51	5.30	6.27	3.63	4.39	5.00	6.21
Fish oil	4.60	4.80	5.10	5.90	6.40	4.80	5.40	5.90	6.70
Amino acid	0.00	0.12	0.25	0.47	0.48	0.12	0.25	0.47	0.47
Taurine	0.00	1.00	1.00	1.00	1.00	1.00	1.00	1.00	1.00
Vitamin premix^c^	2.00	2.00	2.00	2.00	2.00	2.00	2.00	2.00	2.00
Mineral premix^d^	1.00	1.00	1.00	1.00	1.00	1.00	1.00	1.00	1.00
Attractant^e^	1.00	1.00	1.00	1.00	1.00	1.00	1.00	1.00	1.00
Cholesterol	0.00	1.00	1.00	1.00	1.00	1.00	1.00	1.00	1.00
Others^f^	4.00	4.00	4.00	4.00	4.00	4.00	4.00	4.00	4.00
Analytical composition (dry matter, %)									
Energy (kJ g^−1^)	20.19	20.24	20.27	20.38	20.49	20.15	20.15	20.11	20.20
Crude protein	50.00	50.00	50.00	50.00	50.00	50.00	50.00	50.00	50.17
Crude lipid	12.19	12.79	12.49	12.69	12.60	12.73	12.67	12.51	12.65

*FM* diet fish meal, *SBM15* replacement of 15% fish meal protein by soybean meal protein, *SBM30* replacement of 30% fish meal protein by soybean meal protein, *SBM45* replacement of 45% fish meal protein by soybean meal protein, *SBM60* replacement of 60% fish meal protein by soybean meal protein, *FSBM15* replacement of 15% fish meal protein by fermented soybean meal protein, *FSBM30* replacement of 30% fish meal protein by fermented soybean meal protein, *FSBM45* replacement of 45% fish meal protein by fermented soybean meal protein, *FSBM60* replacement of 60% fish meal protein by fermented soybean meal protein

^a^Supplied by Great seven Bio-Tech (Qingdao, China); fish meal, crude protein, 73.77%, crude lipid, 7.58%; soybean meal, crude protein, 52.97%, crude lipid, 2.12%; wheat meal, crude protein, 17.82%, crude lipid, 2.24%; wheat gluten meal, crude protein, 83.31%, crude lipid, 1.75%

^b^Fermented soybean meal got from soybean meal fermented with *Shewanella* sp. MR-7, crude protein, 54.70%, crude lipid, 1.66%

^c^Vitamin premix (mg kg^−1^ diet): retinal palmitate, 32; cholecalciferol, 5; DL-α-tocopherol acetate, 240; menadione, 10; thiamin-HCl, 25; riboflavin, 45; pyridoxine–HCl, 20; cyanocobalamin, 10; D-calcium pantothenate, 60; amine nicotinic acid, 200; folic acid, 20; biotin, 60; mesoinositol, 800; ascorbyl polyphosphate (contained 35% ascorbic acid), 2000; microcrystalline cellulose, 16,473

^d^Mineral premix (mg kg^−1^ diet): MgSO_4_·7H_2_O, 1200; CuSO_4_·5H_2_O, 10; FeSO_4_·H_2_O, 80; ZnSO_4_·H_2_O, 50; MnSO_4_·H_2_O, 45; CoCl_2_·6H_2_O (1%), 50; Na_2_SeO_3_ (1%), 20; calcium iodine, 60; zeolite, 8485

^e^Attractant:betaine:dimethyl-propiothetin:glycine:alanine:5-phosphateinosine = 4:2:2:1:1

^f^Others (10 g kg^−1^ diet): soy lectithin, 2.00; monocalcium phosphate, 1.00; choline chloride, 0.30; Yttrium oxide, 0.10; calcium propionic acid, 0.05; ethoxyquin, 0.05; sodium alginate, 0.50

### Feeding trial and sampling

Juvenile turbots were purchased from a fish rearing farm (Yantai, China). Experiments were done in a flowing water system of Yi Haifeng Aquatic Product CO. Ltd (Qingdao, China). Before the start of the feeding trial, all the fish were acclimated to the culture system for 2 weeks by feeding the commercial diets. After being fasted for 24 h, juvenile turbots with initial weight at 7.57 ± 0.03 g were randomly assigned to 27 experimental fiber glass tanks (30 fish/tank) with each experimental diet assigned to three tanks randomly. Fish were manually fed twice daily at 7:00 and 19:00 till apparent satiation for 79 days.

At the end of 79-day feeding trial, all experimental fish were anesthetized with eugenol (1: 10,000, Shanghai Reagent Co., Shanghai, China). The total number and body weight of the fish in each tank were measured. Five fish from each tank were randomly sampled and stored at − 20 °C for whole body composition analysis. At about 8 h after feeding, only fish with digesta throughout the intestinal tract were sampled for further analysis to ensure intestinal exposure to the diets. The intestine was removed, cleared of any contents, and rinsed several times with ice-cold PBS. The whole intestines and distal intestines of four fish from each tank were sampled separately and stored at − 80 °C for digestive enzymes and quantitative real-time polymerase chain reaction (qRT-PCR) analyses. The entire intestines of another three fish from each tank were immersed in Bouin’s fixative solution and then transferred into 70% ethanol after 24 h for histological evaluation. Moreover, fecal samples were collected from each tank using an automatic fecal collector by siphoning 5 h after feeding from the fifth week of feeding trial. Collected fecal samples were stored at − 20 °C prior to digestibility determination.

For intestinal microbiome analyses, another six fish from each tank were sacrificed and sampled as previously described with modifications [71]. Briefly, the exterior of fish was decontaminated with 70% ethanol, after which the abdomen was dissected with sterile anatomic tools near an alcohol burner and the whole intestine was aseptically removed and opened longitudinally. The contents were removed by mechanical force with forceps. After rinsing the evacuated gut several times with sterile PBS, the intestinal mucosa from the whole intestine was scraped with a sterile scalpel and collected in sterile 1.5 mL tubes. The mucosae of six fish from each tank were pooled as one sample and three samples per dietary group were obtained. All the samples were immediately frozen in liquid nitrogen, and thereafter stored at − 80 °C.

### Biochemical analyses

The glycinin and β-conglycinin of SBM and FSBM were measured by competitive enzyme-linked immunosorbent assay (ELISA) as previously described [81]. Trypsin inhibitors activity was measured using benzoyl-dl-arginine-*p*-nitroanilide (BAPA) method [82]. Raffinose and stachyose were determined using high-performance liquid chromatography (HPLC) as previously described [83]. To measure the apparent digestibility of the diets, the Y_2_O_3_ content in the fecal samples and diets were determined by Inductively Coupled Plasma-atomic Emission SMectrophotometer (ICP-OES, VISTA-MPX) after an acid digestion with perchloric acid [84]. Moisture, crude protein, crude lipid and energy of the diets, ingredients and fish samples were determined using standard method [80]. For digestive enzyme assays, intestine samples were homogenized in ice-cold normal saline and centrifuged at 4000*g* for 20 min at 4 °C to collect the supernatant. The trypsin, amylase and lipase activities were determined as described before [85] using enzymatic assay kits (Jiancheng Bioengineering Institute, China).

### Intestinal histological analyses

After fixation in Bouin’s solution, the distal intestine tissue samples were transferred into 70% ethyl alcohol. Segments of the fixed tissues were dehydrated in a series dilution of ethanol solutions, equilibrated in xylene and embedded in paraffin. Sections (~ 7 μm) were mounted onto albumin coated slides and stained with hematoxylin and eosin (H&E). The slides were observed under an imaging microscope (Olympus, DP72, Nikon, Japan). The ratio (R) between the villi height (VH) and the lumen diameter (LD) of the gut was measured [R = VH/LD, arbitrary units (AU)]. VH of H&E stained hindgut section was determined by measuring about 8–10 well-oriented villus in each sample. A high R value indicates high villi height.

### Quantitative real-time polymerase chain reaction (qRT-PCR)

The expression profiles of mucin-2 (MUC-2) gene, inflammatory marker genes of interleukin-1 beta (IL-1β), tumour necrosis factor alpha (TNF-α), transforming growth factor beta1 (TGF-β1) and tight junction-related genes of zonula occluden-1 (ZO-1) transcript variant 1, occludin and tricellulin were determined using quantitative real-time PCR (qRT-PCR). Total RNA was extracted from distal intestine tissue samples (12 samples/treatment) using TRIzol Reagent (Invitrogen, Carlsbad, CA). High quality RNA with a 260/280-nm absorbance ratio of 1.8–2.0 was reversely transcribed to cDNA by the Primer ScriptTM RT reagent Kit (Takara, China). The qRT-PCR reactions were carried out in 25 μL reaction volume according to previous report [18]. The thermal profile was 95 °C for 20 s, followed by 39 cycles of 95 °C for 5 s, 57–58 °C for 30 s, and 72 °C for 30 s. Three replicate extractions were performed for each sample. To calculate the expression levels of target genes, values were normalized to β-actin (the house-keeping gene), as no expression changes of β-actin were observed in distal intestines among different treatments. The gene expression levels were calculated by 2^−ΔΔCT^ method [86]. The data were reported as fold increase of the control (FM). All primer sequences of target genes were listed in Table 7.

**Table 7:** Sequences of primers used in this study for qRT-PCR

Target gene	Forward primer (5′–3′)	Reverse primer (5′–3′)	GenBank accession number
TNFα	CCCTTATCATTATGGCCCTT	TCCGAGTACCGCCATATCCT	FJ654645.1
IL-1β	TACCTGTCGTGCCAACAGGAA	TGATGTACCAGTTGGGGAA	AJ295836.2
TGFβ1	CTGCAGGACTGGCTCAAAGG	CATGGTCAGGATGTATGGTGGT	KU238187
MUC-2	GTTGGTGCAGCCGCATAG	CACTGGACGCTGGGAATG	KU238186
ZO-1 transcript variant 1	AGAGAACCTGTCACTGATAGATGC	CTGTCGGAATTGTTGCCTGATG	KU238184
Occludin	ACTGGCATTCTTCATCGC	GGTACAGATTCTGGCACATC	KU238182
Tricellulin	GCCTACATCCACAAAGACAACG	TCATTCCCAGCACTAATACAATCAC	KU238183
β-Actin	GCTGTCTTCCCTTCTATCGTCG	TCCATGTCATCCCAGTTGGTC	AY008305.1

*TNF-α* tumor necrosis factor-α, *IL-1β* interleukin-1β, *TGF-β1* transforming growth factor-β1, *MUC-2* mucin 2, *ZO-1* zonula occluden-1

### Intestinal microbiome profiling

The bacterial DNA from intestinal mucosa samples was extracted using the QIAamp DNA Stool Mini Kit (Qiagen, Hilden, Germany). The quantity and purity of the DNA were assessed using a Nano Drop^®^2000 spectrophotometer (Thermo Fisher Scientific, USA). The integrity of DNA was monitored on 1% agarose gels. Then, DNA was diluted to 1 ng μL^−1^ using sterile water and 16S rRNA genes of distinct regions (16SV4) were amplified using specific primer pair 515F (5′-GTGCCAGCMGCCGCGG-3′) and 806R (5′-GGACTACHVGGGTWTCTAAT-3′) with the barcode. All PCR reactions were carried out with Phusion^®^ High-Fidelity PCR Master Mix (New England Biolabs). Thermal cycling consisted of initial denaturation at 98 °C for 1 min, followed by 30 cycles of denaturation at 98 °C for 10 s, annealing at 50 °C for 30 s, and elongation at 72 °C for 30 s. PCR products from all samples were mixed in equidensity ratios and purified using the GeneJET™ Gel Extraction Kit (Thermo Scientific). Sequencing libraries were generated using Ion Plus Fragment Library Kit 48 rxns (Thermo Scientific) according to manufacturer’s recommendations. The library quality was evaluated on the Qubit@ 2.0 Fluorometer (Thermo Scientific). Finally, the library was sequenced on an Ion S5™ XL platform and 400 bp single-end reads were generated.

Single-end reads was assigned to samples based on their unique barcode and truncated by cutting off the barcode and primer sequence. Quality filtering on the raw reads were performed under specific filtering conditions to obtain the high-quality clean reads according to the Cutadapt (v1.9.1) [87] quality-controlled process. The reads were compared with the reference database (Silva database) [88] using UCHIME algorithm [89] to detect chimera sequences, and then the chimera sequences were removed [90]. Then the Clean Reads were finally obtained. Sequences analysis was performed by Uparse software (Uparse v7.0.1001) [91]. Sequences with ≥ 97% similarity were assigned to the same operational taxonomic units (OTUs). Representative sequence for each OTU was screened for further annotation. For each representative sequence, the Silva Database (Version 132) [88] was used based on Mothur algorithm to annotate taxonomic information.

In order to study the phylogenetic relationship of different OTUs, and the difference of the dominant species in different samples (groups), multiple sequence alignments were conducted using the MUSCLE software (v3.8.31) [92]. OTUs abundance information was normalized using a standard of sequence number corresponding to the sample with the least sequences. Subsequent analysis of alpha diversity and beta diversity were all performed basing on this output normalized data. Alpha diversity was applied in analyzing complexity of species diversity for samples through 4 indices, including Chao1, ACE, Shannon and Simpson. All the indices in our samples were calculated with QIIME (v1.7.0) and displayed with R software (v2.15.3). Chao1 and ACE were selected to identify community richness and Shannon and Simpson were selected to identify community diversity. Beta diversity analysis of Principal Coordinate Analysis (PCoA) was used to evaluate differences of samples in species complexity. PCoA analysis was displayed by WGCNA package, stat packages and ggplot2 package in R software (v2.15.3). Tukey’s test and wilcox’s test were used to test statistical difference of alpha diversity and beta diversity between treatments.

### Calculations and statistical methods

The following variables were calculated:

$${\text{Survival rate }}({\text{SR}},\% ) = ({\text{final fish number}}/{\text{initial fish number}}) \times 100\% .$$

$${\text{Weight gain rate }}({\text{WGR}},\% ) = ({\text{final body weight}} \,-\, {\text{initial body weight}})/{\text{initial body weight}} \times 100\% .$$

$${\text{Specific growth rate }}({\text{SGR}},\% ) = {\text{Ln }}({\text{final body weight}}/{\text{initial body weight}})/{\text{days}} \times 100\% .$$

$${\text{Feed intake }}\left( {{\text{FI}},\% } \right) = {\text{dry total feed intake}}/[({\text{final total body weight}}\, + \,{\text{initial total body weight}})/ 2]/{\text{days}} \times 100\% .$$

$${\text{Feed efficiency ratio }}\left( {\text{FER}} \right) = {\text{wet weight gain }}\left( {\text{g}} \right)/{\text{dry feed intake }}\left( {\text{g}} \right).$$

$${\text{Protein efficiency ratio}}\left( {\text{PER}} \right) = {\text{wet weight gain }}({\text{g}})/{\text{protein ingested }}({\text{g}}).$$

$${\text{Apparent digestibility coefficients }}({\text{ADC}},\% ) = ( 1- {\text{Y}}_{ 2} {\text{O}}_{ 3} {\text{in the diet}}/{\text{Y}}_{ 2} {\text{O}}_{ 3} {\text{in feces}}\, \times \,{\text{nutrient in feces}}/{\text{nutrient in diets}}) \times 100\% .$$

All statistical evaluations were analyzed using one-way analysis of variance (ANOVA) by the software SPSS 19.0. Prior to the statistical tests, data were examined for homogeneity of variances. Differences between the means were tested by Tukey’s multiple comparison method. Differences were regarded as significant when *P *< 0.05. Data were expressed as mean ± standard error.

## Supplementary information

**Additional file 1: Figure S1.** Bacterial genera in the intestine of turbot used for the microbe isolation in this study.

**Additional file 2: Table S2.** COG function annotation of *Shewanella* sp. MR-7.

**Additional file 3: Table S3.** KEGG pathway of *Shewanella* sp. MR-7.

**Additional file 4: Table S4.** Genes involved in fatty acid metabolism in the genome of *Shewanella* sp. MR-7.

**Additional file 5: Table S5.** The comparisons of quality parameters of FSBM by *Shewanella* sp. MR-7 and other representative strains. **Figure S2.** Rarefaction curves of observed species number for all the intestinal microbiome samples.

**Additional file 6: Table S6.** The relative abundance of intestinal bacterial OTUs at phylum taxonomic levels.

**Additional file 7: Table S7.** The relative abundance of intestinal bacterial OTUs at genus taxonomic levels.

## Data Availability

The strain *Shewanella* sp. MR-7 has been deposited in China General Microbiological Culture Collection Center (CGMCC) under collection number CGMCC 1.17098. The genome sequence data reported in this study are available in the China National Gene Bank (CNGB) Nucleotide Sequence Archive (CNSA: https://db.cngb.org/cnsa, accession number CNP0000499). The microbiome sequence data reported in this study have been deposited in the Genome Sequence Archive (Genomics, Proteomics & Bioinformatics 2017) in BIG Data Center (Nucleic Acids Res 2018), Beijing Institute of Genomics (BIG), Chinese Academy of Sciences, under accession numbers PRJCA001060 that are publicly accessible at http://bigd.big.ac.cn/gsa.

## Abbreviations

FM: fish meal
SBM: soybean meal
FSBM: *Shewanella* sp. MR-7 fermented soybean meal
CDS: coding sequence
COG: Clusters of Orthologus Groups
KEGG: Kyoto Encyclopedia of Genes and Genomes
GO: gene ontology
ELISA: enzyme-linked immunosorbent assay
TCA-N: trichloroacetic acid soluble nitrogen
ANFs: antinutritional factors
SR: survival rate
WGR: weight gain rate
SGR: specific growth rate
FI: feed intake
FER: feed efficiency ratio
PER: protein efficiency ratio
ADC: apparent digestibility coefficients
qRT-PCR: quantitative real-time polymerase chain reaction
VH: villi height
LD: lumen diameter
R: villi height/lumen diameter
TNF-α: tumor necrosis factor-α
IL-1β: interleukin-1β
TGF-β1: transforming growth factor-β1
MUC-2: mucin 2
ZO-1: zonula occluden-1
OTUs: operational taxonomic units
PCoA: principal coordinate analysis
