# Supplementary material for: Improved utilization of soybean meal through fermentation with commensal Shewanella sp. MR-7 in turbot (Scophthalmus maximus L.)
Source: Microb Cell Fact. 2019 Dec 16;18:214. doi: 10.1186/s12934-019-1265-z (PMC6913000; doi:10.1186/s12934-019-1265-z)
Supplement: Supplementary file 1 — Additional file 1: Figure S1. Bacterial genera in the intestine of turbot used for the microbe isolation in this study. [file 12934_2019_1265_MOESM1_ESM.doc]

**
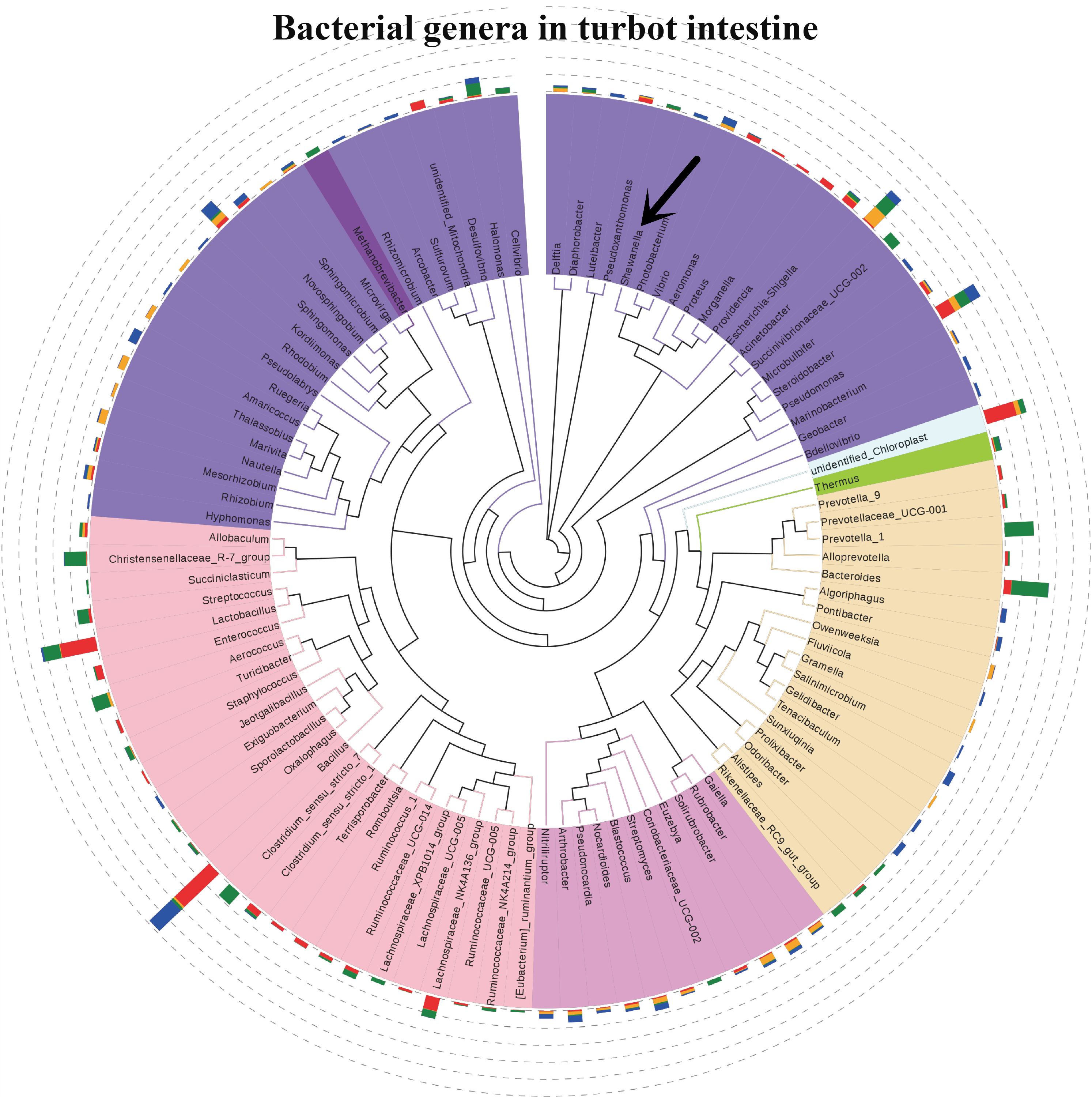
**

**Figure. S1. Bacterial genera in the intestine of turbot used for the microbe isolation in this study.** *Shewanella* was a normal member of the intestinal microbiota in turbot (arrow).
