# Supplementary material for: Improved utilization of soybean meal through fermentation with commensal Shewanella sp. MR-7 in turbot (Scophthalmus maximus L.)
Source: Microb Cell Fact. 2019 Dec 16;18:214. doi: 10.1186/s12934-019-1265-z (PMC6913000; doi:10.1186/s12934-019-1265-z)
Supplement: Supplementary file 5 — Additional file 5: Table S5. The comparisons of quality parameters of FSBM by Shewanella sp. MR-7 and other representative strains. Figure S2. Rarefaction curves of observed species number for all the intestinal microbiome samples. [file 12934_2019_1265_MOESM5_ESM.doc]

**Table S5**

The comparison of quality parameters of FSBM by *Shewanella* sp. MR-7 and other representative strains.

| Fermentation strains | Crude protein | Trypsin Inhibitors | Glycinin | β-conglycinin | Raffinose | Stachyose | Reference |
| --- | --- | --- | --- | --- | --- | --- | --- |
| *Shewanella* sp. MR-7 | +3.20 | -90.19 | -77.36 | -84.52 | -29.25 | -24.25 | This study |
| **Terrestrial strains** |  |  |  |  |  |  |  |
| *Lactobacillus plantarum* P8 | no effects | -87.19 | -77.59 | -55.15 | -99.12 | -84.05 | Wang et al [1] |
| *Lactobacillus acidophilus* | +1.75 | -82.60 | N/A | N/A | no effects | no effects | Chi et al [2] |
| *Rhizopus oligosporus* | N/A | N/A | N/A | N/A | no effects | -83.9 | Egounlety et al [3] |
| *Aspergillus oryzae* | +10.88 | N/A | N/A | N/A | -82.22 | -93.43 | Chen et al [4] |
| *Aspergillus oryzae* AO3042 | +0.34 | -82.00 | N/A | N/A | N/A | N/A | Teng et al [5] |
| *B. subtilis* SB102 | +8.37 | -96.00 | N/A | N/A | N/A | N/A |
| *B. subtilis* KCCM11438P | N/A | -50.00 | -42.00 | -70.00 | N/A | N/A | Seo et al [6] |
| *B. amyloliquefaciens* | +5.32 | N/A | N/A | N/A | N/A | N/A | Medeiros et al [7] |
| 1. *subtilis*10160 | +17.18 | -51.18 | N/A | N/A | N/A | N/A | Dai et al [8] |
| Compound bacteria *(Streptococcus thermophilus*, *B. subtilis* MA139, *Saccharomyces cerevisae)* | +3.37 | N/A | -62.22 | -40.87 | N/A | N/A | Wang et al [9] |
| **Fish derived strains** |  |  |  |  |  |  |  |
| *B. pumillus* SE5 | +15.61 | N/A | -43.67 | -57.51 | N/A | N/A | Rahimnejad et al [10] |
| *Pseudozyma aphidis* ZR1 | +15.42 | N/A | -39.87 | -53.86 | N/A | N/A |

Note: Values in this table mean the increasing rate (%) of crude protein and degradation rate (%) of ANFs in soybean meal after fermentation with *Shewanella* sp. MR-7 and other representative strains listed. ‘No effects’ means that fermentation makes no effects on the corresponding index according to the reference. ‘N/A’ means that the corresponding index is not measured in the reference.

**Reference**

1. Wang L, Zhou H, He R, Xu W, Mai K et al. Effects of soybean meal fermentation by *Lactobacillus plantarum* P8 on growth, immune responses, and intestinal morphology in juvenile turbot (*Scophthalmus maximus* L.). Aquaculture. 2016;464:87-94.
2. Chi C-H, Cho S-J. Improvement of bioactivity of soybean meal by solid-state fermentation with *Bacillus amyloliquefaciens* versus *Lactobacillus* spp. and *Saccharomyces cerevisiae*. LWT-Food Sci Technol. 2016;68:619-625.
3. Egounlety M, Aworh OC. Effect of soaking, dehulling, cooking and fermentation with *Rhizopus oligosporus* on the oligosaccharides, trypsin inhibitor, phytic acid and tannins of soybean (*Glycine max* Merr.), cowpea (*Vigna unguiculata* L. Walp) and groundbean (*Macrotyloma geocarpa* Harms). J Food Eng. 2003;56:249-254.
4. Chen CC, Shih YC, Chiou PWS, Yu B. Evaluating nutritional quality of single stage- and two stage-fermented soybean meal. Asian Australas J Anim Sci. 2010;23:598-606.
5. Teng D, Gao M, Yang Y, Liu B, Tian Z, Wang J. Bio-modification of soybean meal with *Bacillus subtilis* or *Aspergillus oryzae*. Biocatal Agric Biotechnol. 2012;1:32-38.
6. Seo S-H, Cho S-J. Changes in allergenic and antinutritional protein profiles of soybean meal during solid-state fermentation with *Bacillus subtilis*. LWT-Food Sci Technol. 2016;70:208-212.
7. Medeiros S, Xie J, Dyce PW, Cai HY, DeLange K et al. Isolation of bacteria from fermented food and grass carp intestine and their efficiencies in improving nutrient value of soybean meal in solid state fermentation. J Anim Sci Biotechnol. 2018;9:29.
8. Dai C, Ma H, He R, Huang L, Zhu S, Ding Q, Luo L. 2017. Improvement of nutritional value and bioactivity of soybean meal by solid-state fermentation with *Bacillus subtilis*. Lwt-Food Sci Technol 86:1-7.
9. Wang Y, Liu XT, Wang HL, Li DF, Piao XS et al. Optimization of processing conditions for solid-state fermented soybean meal and its effects on growth performance and nutrient digestibility of weanling pigs. Life Sci. 2014;170:91-99.
10. Rahimnejad S, Lu K, Wang L, Song K, Mai K et al. Replacement of fish meal with *Bacillus pumillus* SE5 and *Pseudozyma aphidis* ZR1 fermented soybean meal in diets for Japanese seabass (*Lateolabrax japonicus*). Fish Shellfish Immunol. 2018;84:987-997.


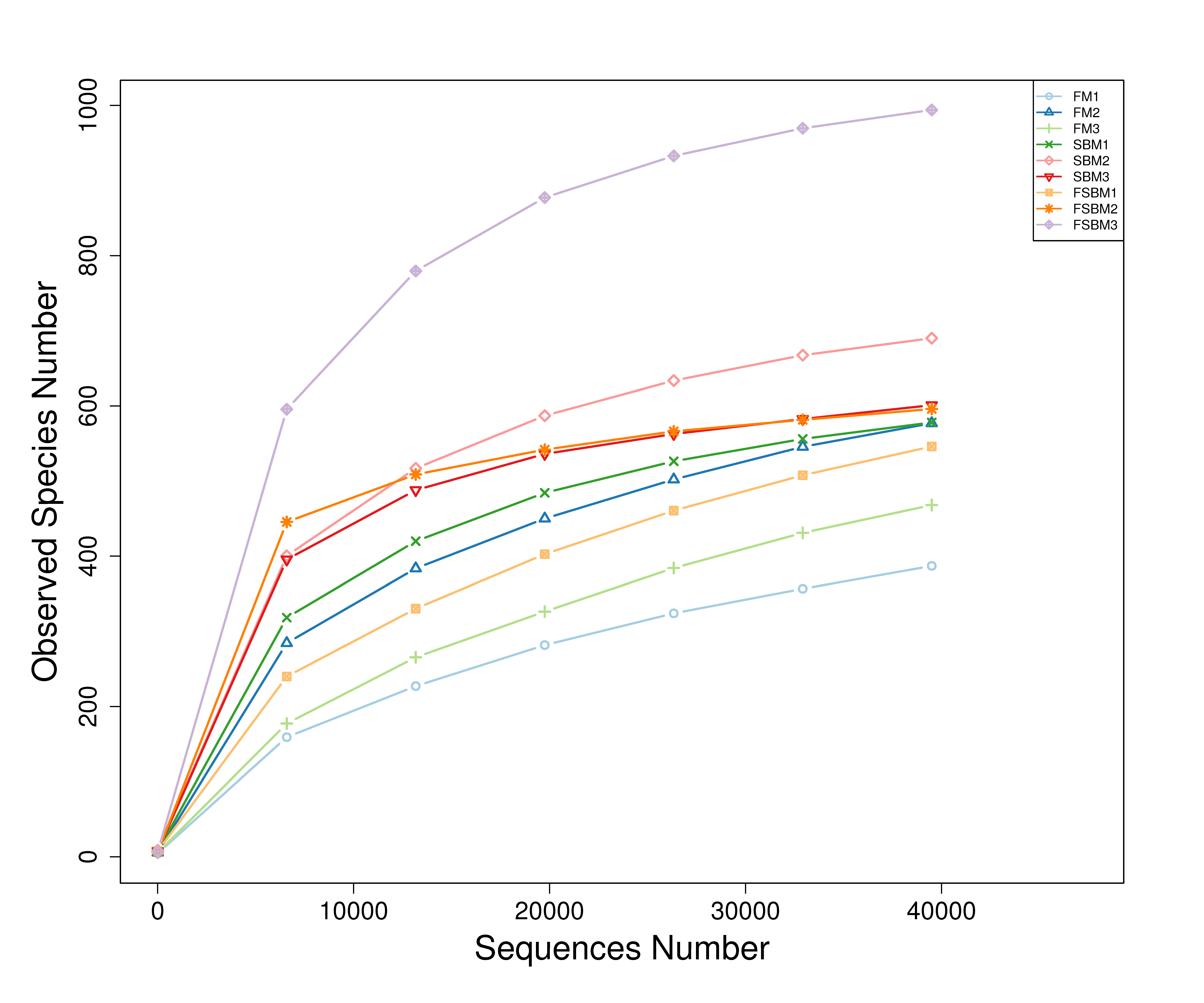
**Figure. S2. Rarefaction curves of observed species number for all the intestinal microbiome samples.** The rarefied curves for observed species number tended to approach the saturation plateau.

Abbreviation: FM1, FM2 and FM3 are three replicates of FM group; SBM1, SBM2 and SBM3 are three replicates of SBM45 group; FSBM1, FSBM2 and FSBM3 are three replicates of FSBM45 group.
